# Symptomatic Dengue Disease in Five Southeast Asian Countries: Epidemiological Evidence from a Dengue Vaccine Trial

**DOI:** 10.1371/journal.pntd.0004918

**Published:** 2016-08-17

**Authors:** Joshua Nealon, Anne-Frieda Taurel, Maria Rosario Capeding, Ngoc Huu Tran, Sri Rezeki Hadinegoro, Tawee Chotpitayasunondh, Chee Kheong Chong, T. Anh Wartel, Sophie Beucher, Carina Frago, Annick Moureau, Mark Simmerman, Thelma Laot, Maïna L’Azou, Alain Bouckenooghe

**Affiliations:** 1 Sanofi Pasteur Asia & JPAC Region, Singapore; 2 Research Institute for Tropical Medicine, Alabang, Muntinlupa City, Philippines; 3 Pasteur Institute Ho Chi Minh City, Ho Chi Minh City, Vietnam; 4 University of Indonesia Medical School, Cipto Mangunkusumo Hospital, Jakarta, Indonesia; 5 Queen Sirikit National Institute of Child Health, Bangkok, Thailand; 6 Disease Control Division, Malaysian Ministry of Health, Putrajaya, Malaysia; 7 Sanofi Pasteur, Marcy L’Etoile, France; 8 Sanofi Pasteur, Global Epidemiology, Lyon, France; Duke-NUS GMS, SINGAPORE

## Abstract

Dengue incidence has increased globally, but empirical burden estimates are scarce. Prospective methods are best-able to capture all severities of disease. CYD14 was an observer-blinded dengue vaccine study conducted in children 2–14 years of age in Indonesia, Malaysia, Thailand, the Philippines, and Vietnam. The control group received no vaccine and resembled a prospective, observational study. We calculated the rates of dengue according to different laboratory or clinical criteria to make inferences about dengue burden, and compared with rates reported in the passive surveillance systems to calculate expansion factors which describe under-reporting. Over 6,933 person-years of observation in the control group there were 319 virologically confirmed dengue cases, a crude attack rate of 4.6%/year. Of these, 92 cases (28.8%) were clinically diagnosed as dengue fever or dengue hemorrhagic fever by investigators and 227 were not, indicating that most symptomatic disease fails to satisfy existing case definitions. When examining different case definitions, there was an inverse relationship between clinical severity and observed incidence rates. CYD14’s active surveillance system captured a greater proportion of symptomatic dengue than national passive surveillance systems, giving rise to expansion factors ranging from 0.5 to 31.7. This analysis showed substantial, unpredictable and variable under-reporting of symptomatic dengue, even within a controlled clinical trial environment, and emphasizes that burden estimates are highly sensitive to case definitions. These data will assist in generating disease burden estimates and have important policy implications when considering the introduction and health economics of dengue prevention and control interventions.

## Author Summary

Dengue is a mosquito-borne, viral febrile disease transmitted between humans in most of the tropical and sub-tropical world. In recent years, an increasing number of cases has been widely reported. However, understanding the full disease burden remains a topic of public health research. One reason for under-reporting is that severe episodes are more likely to be captured in routine surveillance statistics, and mild episodes unreported/unrecognized. We re-analyzed data from the control arm of a dengue vaccine clinical trial in five Asian countries. The trial captured dengue incidence rates following active surveillance and virological confirmation, and we compared those with incidence rates from the passive surveillance system. As expected, the active surveillance system captured many more cases of symptomatic dengue than routine systems. Of virologically confirmed dengue in the clinical trial, only ~29% were diagnosed by investigators as dengue, indicating there is a significant disease burden excluded from existing case definitions and diagnostic practices. The analysis confirmed that dengue is under-reported, by different magnitudes, in these Asian countries. Case definition is an important determinant of burden. These findings are important when considering the health economics and public health impacts of new prevention and control tools.

## Introduction

Dengue is a viral disease transmitted between humans by *Aedes* mosquitoes throughout the tropical and subtropical world. Infection may be asymptomatic, or can result in a spectrum of clinical disease including self-limiting fever with manifestations of varying severity (classical dengue fever; DF) progressing to life-threatening dengue hemorrhagic fever (DHF) and dengue shock syndrome (DSS). [1] While this classification remains in clinical use in some countries, a new system was proposed by the World Health Organization (WHO) in 2009 primarily to improve triage and clinical management, and to capture warning signs of potentially severe dengue episodes. [1,2] Disease prevention efforts with mosquito control have been largely unsuccessful and recent decades have witnessed increased disease frequencies and expanded ranges of transmission. [3,4] Dengue is now endemic in over 120 countries worldwide, with almost half of the global population at risk. [3,5]

Approximately 75% of this at-risk population resides within the Asia Pacific region, where the primary vectors (*Aedes aegypti* and *Ae*. *albopictus)* and dengue virus have become widely dispersed over recent decades following a number of social, environmental, and demographic changes. [6,7] Multiple dengue virus serotypes co-circulate and the disease constitutes a leading cause of hospitalization and death in some countries. [8] In the midst of this expansion, and possibly due to it, reliable dengue disease burden estimates are uncommon. [3,9] Passive national dengue surveillance systems are designed to detect outbreak activity rather than describe burden. [10] More reliable estimates are required to guide disease control programs, allow rational allocation of resources, and assess the impact of new interventions such as dengue vaccination. Accordingly, estimating the true disease burden constitutes one of the WHO’s three objectives in the 2012 *Global Strategy for Dengue Prevention and Control 2012–2020*. [3]

In most scenarios, national surveillance systems underestimate disease burden due to the non-specific clinical presentation of dengue; unavailability and limitations of confirmatory diagnostic tests; and health system issues that result in incomplete reporting. [11] Under-estimation is typically most severe in the milder manifestations of illness, and is a function both of under-ascertainment and under-reporting. [12] In recent years, several methods have been used to improve the accuracy of historical global disease burden estimates of approximately 100 million infections/year. [3,13] These include empirical methods where overlapping data sources enable estimation of cases missed (capture-recapture studies); expert consensus-based approaches; statistical and/or cartographic methods incorporating dengue occurrence data or their covariates; regression methods to estimate unknown variables; and derivations from seroprevalence data. [9,14–16] Notably, a 2013 study by Bhatt *et al*. used a cartographic modeling approach combining demographic and epidemiological data, adjusted for clinical severity and determinants of dengue incidence, to estimate a global burden of 390 million (95% credible interval: 284–528 million) infections in 2010, of which 96 million (67–136 million) were symptomatic. [9] It has been estimated that 70% of cases and >50% of the economic burden of dengue are in Asia. [9,17]

Prospective cohort studies utilizing active surveillance can yield more accurate estimates of symptomatic disease than passive surveillance systems. [18] Resulting incidence rates (IRs), when compared with data from routine surveillance systems, can describe the extent of under-estimation, expressed as multiplication or expansion factors (EFs). [12,19] In Cambodia, Thailand, and the Philippines, individual studies using these methods calculated EFs for dengue of between 7.2 and 9.1. [20,21] A review using data from all WHO regions found dengue EFs in Asia of up to 126, with significant variation among countries and over time resulting from different underlying epidemiology, surveillance practices, and comparative study design. [19]

Dengue vaccine clinical trials are conducted with a high degree of operational integrity and produce data closely resembling those from active epidemiological studies. Subjects allocated to the control group do not receive dengue vaccine, so incidence data from these individuals can be interpreted as an observational dengue cohort. [22] CYD14 was an observer-blinded dengue vaccine study conducted in 2011–2013 in 10,275 children aged 2–14 years in Indonesia, Malaysia, Thailand, the Philippines, and Vietnam. [23] Each of these countries conducts passive routine dengue surveillance, sometimes using different case definitions and different reporting, laboratory, and diagnostic practices. [10,11] (described in S1 File).

Dengue epidemiological data from CYD14 and its Latin American sister, CYD15, were recently described by L’Azou *et al*., allowing comparison across countries of data collected using standardized, active methods. [24] Here, we exploit the comprehensive dataset to further explore dengue incidence in the CYD14 control group according to different clinical endpoints (in addition to the primary clinical endpoint of the efficacy trial) to examine the relationship between burden and severity in five Asian countries. We also made comparisons with national surveillance reports to estimate EFs for symptomatic dengue of different clinical severities, from which broader burden estimates can be inferred.

## Materials and Methods

### Ethics statement

This was a secondary analysis using records from a vaccine clinical trial. The original clinical trial which generated the data (ClinicalTrials.gov number NCT01373281) underwent ethics committee approval of the protocol, amendments, consent, and assent forms. [23] Parents or legal guardians provided informed consent before participation, and written assent was obtained from older children, in compliance with the regulations of each country. All data were analyzed anonymously.

### CYD14 study design and data

**Fig 1:**
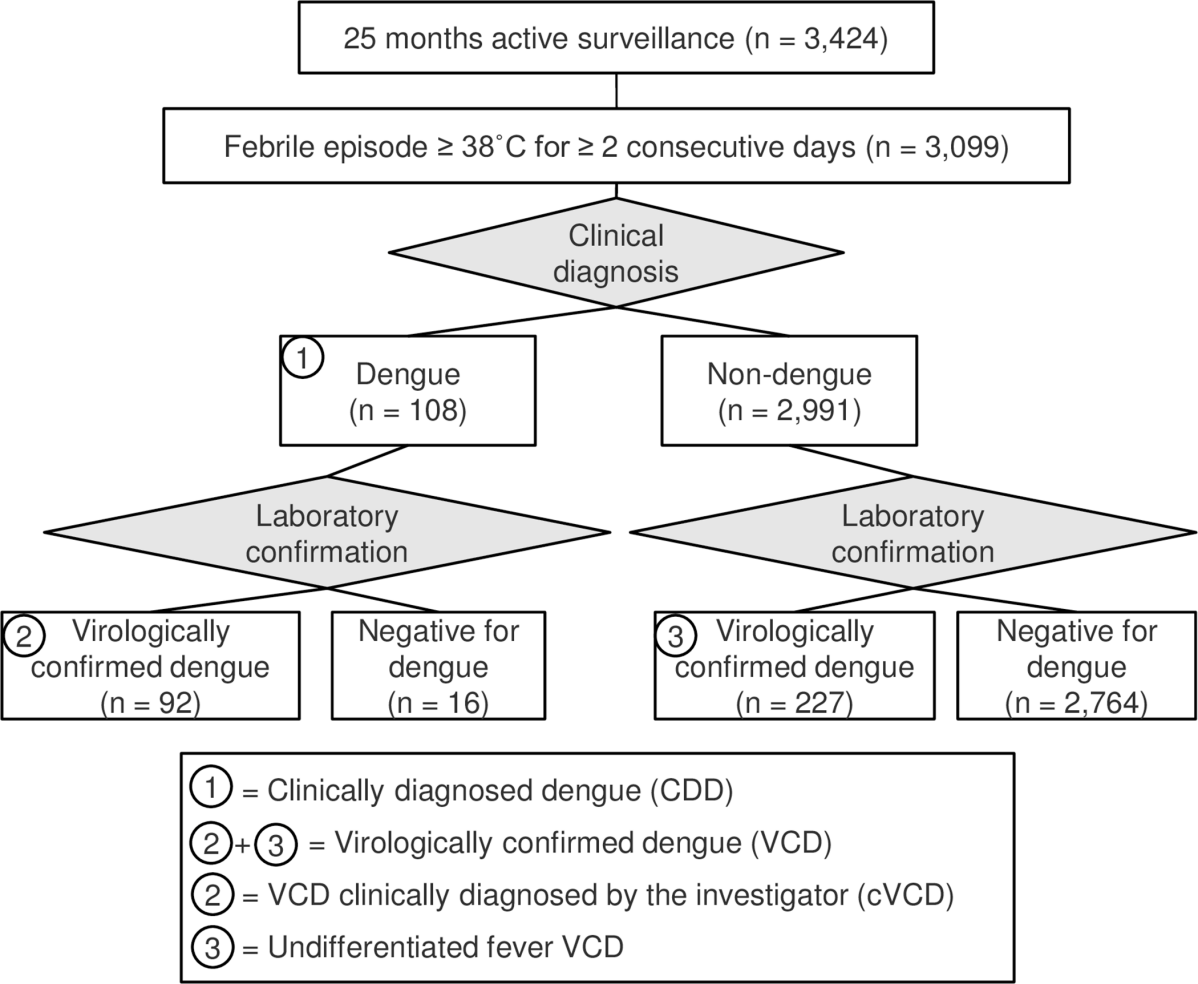
CYD14 study flow chart and source of each case definition. Control arm subjects were actively followed for 25 months to detect episodes of fever ≥38°C for ≥ 2 consecutive days. Febrile episodes were recorded and clinically diagnosed as dengue based on 1997 WHO guidelines, or an alternative etiology. Irrespective of clinical diagnosis, serum samples were taken for virological confirmation of dengue by detection of NS1 antigen by immunoassay and viral RNA by RT-PCR. A positive result for either laboratory test was considered confirmatory of dengue. Clinically diagnosed dengue (CDD): all episodes that were clinically diagnosed as dengue, irrespective of virological confirmation. VCD: all virologically confirmed dengue episodes, irrespective of clinical diagnosis. cVCD: all VCD episodes that were also clinically diagnosed as dengue. UF-VCD: all VCD episodes that were not clinically diagnosed as dengue.

CYD14 (CT.gov identifier NCT01373281) was an observer-masked, randomized, controlled, multicenter, phase 3 dengue vaccine trial in Indonesia (3 study centers), Malaysia (2 study centers), the Philippines (2 study centers), Thailand (2 study centers), and Vietnam (2 study centers) and has been described previously. [23] There was ethics committee approval of the protocol, amendments, consent, and assent forms. Parents or legal guardians provided informed consent before participation, and written assent was obtained from older children, in compliance with the regulations of each country. Briefly, children aged 2–14 years were randomly assigned to receive three injections of a recombinant, live-attenuated, tetravalent dengue vaccine (CYD-TDV), or placebo, at 0, 6, and 12 months. Participants were followed up actively for a total of 25 months and episodes of fever ≥ 38°C on ≥ 2 consecutive days were recorded and clinically diagnosed as DF or DHF based on 1997 WHO guidelines (Fig 1). Concurrent with and irrespective of clinical diagnosis, serum samples were taken for virological confirmation of dengue by detection of NS1 antigen by ELISA and dengue viral RNA by RT-PCR. A positive result for either laboratory test was considered virological confirmation of acute dengue infection. This allowed febrile individuals to be grouped into four case definitions according to their clinical diagnosis and laboratory results: 1) clinically diagnosed dengue (CDD) was diagnosed by the investigator as dengue, irrespective of the laboratory result; 2) virologically confirmed dengue (VCD) was a dengue virological laboratory confirmation, irrespective of the clinical diagnosis; 3) clinical VCD (cVCD) was clinically diagnosed by the investigator as dengue and accompanied by laboratory confirmation of dengue infection; 4) undifferentiated fever VCD (UF-VCD) was laboratory confirmation of dengue infection but was not diagnosed as dengue by the investigator. Detailed case report forms were completed for each febrile episode, including whether subjects required hospitalization. This manuscript describes results of a secondary analysis of anonymous data from this vaccine clinical trial.

### National dengue surveillance, population data and incidence rates

Sub-national passive dengue surveillance data from districts, provinces, or cities (hereafter referred to as “geographical units”) encompassing each clinical trial center were retrieved from official government surveillance websites for Thailand [25] and Jakarta, Indonesia, [26] or from personal communications with trial investigators, or sub-national health authorities in Malaysia [27], Indonesia, the Philippines, and Vietnam [28]. Dengue cases of any severity were pooled for the period of time during which CYD14 was active in that country.

Age-specific population data for sites were retrieved from census or other official records for each geographical unit. [29–33] Populations at the mid-point of the study were estimated by applying national-level population growth factors. Where surveillance or census data lacked age-stratifications (Vietnam and Indonesia for census; Malaysia and Thailand for monthly age-specific surveillance data), we assumed age-distributions of populations/cases were proportional to those at the national level. Average annual IRs were calculated for each country by pooling data from all geographical areas participating in the study, expressed as cases/100,000 population/year, as:

$$Average\;annual\;incidence\;rate=\frac{\frac{N_{c}}{T_{m}}}{\bar{{Population}_{ji}}}*12$$

Where *N_c_* is the number of cases reported to the surveillance system over the study observation period; *T_m_* is the duration of the study in each country, in months; and $\bar{{Population}_{ji}}$ is the average population size over the study period.

### Calculating CYD14 incidence densities

Site- and age-weighted incidence densities (IDs) were calculated by direct standardization for each country to correct for the fact that the age and geographic distributions of study populations were different from those in the geographic units from which they were drawn. [34] Study populations were divided into three age groups according to their age when they contributed time to the study: < 5 years; 5 −< 10 years; and > 10 years (all were aged <15 years at enrollment). Crude age-specific IRs were calculated for each age group and each center by dividing the number of cases satisfying each case definition by the number of person-years (p/y) of observation. These crude IRs were to match the demographics from CYD14 with those of each geographical area, resulting in age- and site-adjusted IDs aligned with the census populations at the country level. [34] Results were presented as cases/100,000 p/y. Standardized 95% confidence intervals (CIs) were calculated based on the gamma distribution using SAS 9.4 (SAS Institute, Cary, NC).[35]

### Expansion factors and case definitions

Expansion factors were calculated by dividing the adjusted ID captured during CYD14 for each case definition by the IRs reported by the national passive surveillance systems at each geographical unit. For calculating 95% CIs, IRs from surveillance systems were considered known data without variability. [34]

Descriptive exploratory statistical analysis was performed on the ability of each case definition to identify symptomatic dengue cases; the proportion hospitalized; and the duration of clinical symptoms, fever, and hospitalization, for each. Using VCD as the gold standard diagnosis of dengue following a febrile episode, positive predictive value, negative predictive value, sensitivity, and specificity were calculated for clinical diagnosis of dengue disease with their 95% CIs according to the efficient-score method [36]. Data used for the analyses described above are provided in S1 and S2 Tables.

## Results

### VCD and CDD in the CYD14 cohorts

Between June and December, 2011, 3,424 children were enrolled in the control arm of the CYD14 study (Table 1). The demographics of the subjects have been reported elsewhere. [23] The period of observation was 6,933 p/y, during which there were 3,099 febrile episodes tested for dengue, of which 319 (10.3%) were VCD. This proportion in each country varied between 6.3% (Malaysia) and 12.3% (Indonesia). The overall crude annual VCD attack rate was 4.6%, varying from 2.2% (Malaysia) to 6.6% (Philippines). A total of 108 cases satisfied the CDD definition and 227 satisfied the UF-VCD definition (underlying data in S1 Table).

**Table 1:** Number (n) and proportion of subjects experiencing episodes satisfying different case definitions in the CYD14 control arm, June 2011 –December 2013.

Country	Subjects injected n	Febrile episodes, n	Person-years followed, n	VCD episodes, n (% of fevers)	cVCD episodes, n (% of fevers)	CDD episodes^1^, n (% of fevers)	UF- VCD^2^, n (% of fevers)	Proportion of VCD clinically diagnosed as dengue^3^, %	Crude VCD attack rate (%), %
Indonesia	623	357	1232	44 (12.3)	26 (7.3)	33 (9.2)	18 (5.0)	59.1	3.6
Malaysia	465	332	937	21 (6.3)	9 (2.7)	11 (3.3)	12 (3.6)	42.9	2.2
Philippines	1166	1420	2370	156 (11.0)	16 (1.1)	19 (1.3)	140 (9.9)	10.3	6.6
Thailand	392	388	792	47 (12.1)	35 (9.0)	36 (9.3)	12 (3.1)	74.5	5.9
Vietnam	778	602	1602	51 (8.5)	6 (1.0)	9 (1.5)	45 (7.5)	11.8	3.2
Totals	3,424	3,099	6,933	319 (10.3)	92 (30)	108 (3.5)	227 (7.3)	28.8	4.6

n, number of subjects or events; VCD, virologically confirmed dengue. cVCD, clinically diagnosed and virologically confirmed dengue; CDD, clinically diagnosed dengue; UF-VCD, virologically confirmed dengue not diagnosed as dengue. Crude attack rates are VCD cases/person-years followed.

^1^Total episodes that were clinically diagnosed dengue (CDD).

^2^VCD episodes accompanied by a clinical diagnosis other than dengue.

^3^cVCD/VCD (i.e. sensitivity).

Of the 319 VCD cases, 25 (7.8%) were clinically diagnosed as DHF and 67 (21.0%) as DF, giving a total of 92 (28.8%) cases of cVCD. This proportion of VCD correctly diagnosed varied widely among the countries, from 10.3% (Philippines) to 74.5% (Thailand). In addition to the 25 clinical diagnoses of DHF in the VCD group, there were an additional 4 DHF diagnoses which were not virologically confirmed. Only one DHF diagnosis was in a subject aged <5.

### Incidence of dengue in the cohorts compared to national routine surveillance estimates

There was significant heterogeneity in average IRs observed between countries over the duration of the clinical trial. The IRs for all dengue cases (per 100,000 p/y) reported to national routine surveillance systems for the study locations during the study period varied by country from: 64.7 (Malaysia); 263 (Indonesia); 497 (Thailand); 509 (Vietnam); and 954 (Philippines) (Table 2). Adjusted dengue IDs (per 100,000 p/y) in the CYD14 study were considerably higher than the rates captured by the national systems and varied according to the case definition used. IDs were highest for VCD (range: 2,048 [Malaysia] to 10,960 [Philippines]), and were followed by the IDs for UF-VCD (range: 1,192 [Indonesia] to 10,290 [Philippines]), CDD (range: 701 [Philippines] to 4,383 [Thailand]), and finally cVCD (range: 261 [Vietnam] to 4,262 [Thailand]). Surveillance data and corresponding incidence rates provided in S2 Table.

**Table 2:** Dengue incidence rates [and 95% CIs] from routine surveillance systems and adjusted incidence densities of disease according to different case definitions from the CYD14 study.

Country	Average IR from routine surveillance system	CYD14			
		VCD, adjusted ID	cVCD, adjusted ID	CDD, adjusted ID	UF-VCD, adjusted ID
Indonesia	262.9	3,017 [1,951–4,542]	1825 [996, 3,153]	2,479 [1,483, 3,963]	1,192 [602, 2,269]
Malaysia	64.7	2,048 [1,099, 3,720]	671 [288, 1,851]	777 [367, 1,961]	1,377 [567, 2,993]
Philippines	954.4	10,960 [8,673, 13,620]	677 [262, 1,439]	701 [281, 1,461]	10,290 [8,055, 12,890]
Thailand	496.7	5,938 [4,273, 8,059]	4,262 [2,914, 6,055]	4,383 [3,015, 6,194]	1,676 [808, 3,065]
Vietnam	509.2	2,784 [1,813, 4,238]	261 [94, 1,083]	840 [156, 2,371]	2,523 [1,580, 3,964]

Incidence rates and densities are in cases/100,000 person-years. Incidence rates for routine surveillance calculated from total number of cases over the period of study and study mid-point populations. IR, incidence rate; VCD, virologically confirmed dengue; ID, incidence density; CI, confidence interval; cVCD, clinically diagnosed and virologically confirmed dengue; CDD, clinically diagnosed dengue; UF-VCD, virologically confirmed dengue not diagnosed as dengue.

### Expansion factors

EFs varied according to case definitions. They were 5.5−31.7 for VCD, 0.5−10.4 for cVCD, and 0.7−12.0 for CDD (Table 3). These factors varied widely but tended to be lowest in Vietnam and highest in Malaysia. The incidence rates of dengue reported to the routine surveillance system appeared to be important determinants of EF: the highest EF (31.7) was observed in Malaysia (with the lowest reported IR) and the Philippines (with low EFs) reported the highest IRs in passive surveillance.

**Table 3:** Expansion factors for VCD, cVCD, and CDD over the active phase of the CYD14 study.

Country	VCD [95% CI]	cVCD [95% CI]	CDD [95% CI]
**Indonesia**	11.5 [7.4, 17.3]	6.9 [3.8, 12.0]	9.4 [5.6, 15.1]
**Malaysia**	31.7 [17.0, 57.5]	10.4 [4.5, 28.6]	12.0 [5.7, 30.3]
**Philippines**	11.5 [9.1, 14.3]	0.7 [0.3, 1.5]	0.7 [0.3, 1.5]
**Thailand**	12.0 [8.6, 16.2]	8.6 [5.9, 12.2]	8.8 [6.1, 12.5]
**Vietnam**	5.5 [3.6, 8.3]	0.5 [0.2, 2.1]	1.7 [0.3, 4.7]

CI, confidence interval; VCD, virologically confirmed dengue CDD, cVCD, clinically diagnosed and virologically confirmed dengue; CDD, clinically diagnosed dengue.

### Hospitalization and symptoms

Overall, 126 (4.1%) of the acute febrile episodes in the cohort were hospitalized (Table 4). For the individual countries, this proportion varied between 1.2% (Vietnam) and 8.8% (Thailand). Hospitalization rates varied according to local standard of care (i.e., laboratory and clinical diagnosis): 61 (19.1%) of the 319 VCD episodes; 62 (57.4%) of the 108 CDD cases; and 24 (96.0%) of the 25 VCD cases diagnosed as DHF, were hospitalized. These proportions varied between countries. Clinical dengue diagnosis appeared to be an important determinant of hospitalization. The median duration of clinical symptoms was 5.0 days [min; max: 2.0; 38.0] for cases of UF-VCD; 6.0 [2·0; 38·0] for VCD and 8.0 [2.0; 31.0] for CDD. Median durations of fever were 3.0 [2.0; 11.0], 3·0 [2·0; 11·0] and 4.0 [2.0; 9.0)] and hospitalization 4.0 [3.0; 6.0]; 5·0 [2·0; 9·0] and 5.0 [2.0; 9.0)], respectively.

**Table 4:** Number of episodes and hospitalizations in CYD14 study control subjects experiencing acute fever, VCD, CDD, or VCD clinically diagnosed DHF.

Country	Febrile episodes		VCD		CDD		VCD clinically diagnosed DHF	
	n	Number of episodes hospitalized (%)	n	Number of episodes hospitalized (%)	n	Number of episodes hospitalized (%)	n	Number of episodes hospitalized (%)
Indonesia	357	30 (8.4)	44	20 (45.5)	33	21 (63.6)	10	11 (100.0)
Malaysia	332	20 (6.0)	21	8 (38.1)	11	7 (63.6)	1	1 (100.0)
Philippines	1420	35 (2.5)	156	17 (10.9)	19	17 (89.5)	9	10 (100.0)
Thailand	388	34 (8.8)	47	13 (27.7)	36	13 (36.1)	2	2 (100.0)
Vietnam	602	7 (1.2)	51	3 (5.9)	9	4 (44.4)	3	3 (60.0)
**Total**	**3,099**	**126 (4.1)**	**319**	**61 (19.1)**	**108**	**62 (57.4)**	**25**	**24 (96.0)**

n, number of subjects or events; VCD, virologically confirmed dengue, CDD, clinically diagnosed dengue; DHF, dengue haemorrhagic fever.

### Positive and negative predictive values, sensitivity, and specificity of clinical diagnosis

The positive predictive value (PPV) of clinical diagnosis, using VCD as the gold standard, was 85.2% (95% CI 77.1−91.3) and the negative predictive value (NPV) was 92.4% (91.4−93.3). The sensitivity of clinical diagnosis was 28.8% (95% CI 23.9−34.2) and the specificity was 99.4% (99.1−99.7).

## Discussion

We used the control arm of a large, phase 3 efficacy dengue vaccine trial to describe the symptomatic and virologically-confirmed dengue burden in five Asian countries. This permitted comparison of clinical vs. laboratory dengue diagnosis for different classifications of symptomatic dengue identified through active surveillance. Importantly, data were consistently collected according to standardized case definitions and with high-quality virological confirmation, allowing IDs to be measured for different clinical outcomes and in different countries, within a single study. Rates observed in study participants were adjusted to match the populations from which they were sampled.

Discrepancies between dengue clinical and laboratory diagnosis typically find case definitions which are sensitive but lack specificity, particularly in episodes of mild disease. [37–39] Our results showed that virological confirmation was the most sensitive means of identifying dengue disease, capturing approximately 3.5 times more episodes than clinical diagnosis alone, even in this acutely febrile patient population. Clinical diagnosis alone captured only 28.8% of symptomatic cases. Because most passive disease surveillance systems in Asia rely almost entirely on clinical diagnosis, [10,40] it is reasonable to believe a substantial proportion of symptomatic dengue disease is unrecognized and therefore unreported.

The proportion of VCD clinically diagnosed by investigators as dengue varied substantially between countries (range: 10.3%−74.5%), likely resulting from the multifactorial impacts of local clinical guidelines and case definitions that affect diagnostic practices, and variable clinical presentations. Disease severity and clinical manifestation may be affected by factors including circulating viral genotypes; the order and duration between sequential, heterotypic infections; year/season; and subject age [41–43]. Notably, VCD cases appeared to be younger in the Philippines–where dengue was least-frequently diagnosed–than other countries.

Estimates of dengue burden are, in large part, a function of case definition. [9] The active surveillance methods here allowed calculation of IRs according to different case definitions (VCD, cVCD, and CDD) and thus determine EFs for each. The higher rates of VCD captured gave rise to EFs ranging from 5.5 to 31.7, with lower EFs for more specific case definitions of cVCD and CDD. These figures are notable for their variability and emphasize that study and surveillance system methodology and geography are important determinants of under-reporting estimates, as reported elsewhere. [15,19] However, the finding that dengue is under-reported by factors of >30 in some countries and contexts is consistent between these studies. Notable exceptions are the expansion factors <1, observed in Philippines and Vietnam against specific, clinically-diagnosed case definitions (cVCD and CDD): passive surveillance had captured a higher proportion of cases than the active system. There are two likely possibilities: 1) the passive surveillance reported false-positive cases (ie, episodes of febrile, non-dengue disease, thereby increasing the denominator) or, more likely, 2) the active system excluded febrile cases which failed to satisfy case definitions (thereby reducing the numerator). Both scenarios emphasize the heterogeneity of dengue case definitions, surveillance systems and clinical practices, which challenge the generation of consistent burden estimates. Additional complexity has been observed during outbreaks from both over- and under-reporting due to differing levels of disease awareness and/or reporting practices. [44]

A similar analysis was conducted in slightly older Latin American children, focusing on VCD cases and comparing with dengue reported at different levels of the surveillance system (country; state; local). [45] It found lower rates of VCD (from 2,500 cases/100,000 p/y in Mexico to 3,500 in Brazil), and corresponding EFs which varied widely, from 3.5–45.5 (depending on country and comparator), emphasizing that EFs are a complex outcome of local epidemiology, disease awareness, health system characteristics and other factors. [19] Additional analyses of under-reporting according to indicators of socio-demography or dengue awareness, for example, may be illuminating.

Hospitalization was based on local routine practice and rates were shown to be substantial: over 4% of fevers, and over 50% of dengue diagnoses were hospitalized. A clinical diagnosis–rather than virological confirmation–seemed to determine the decision to hospitalize, demonstrated by the successively decreasing incidence and increasing hospitalization rate of episodes of fever; VCD; CDD; and DHF. Interestingly, four cases of clinically diagnosed DHF (13.8% of the total) could not be virologically confirmed, highlighting a possible over-attribution in endemic areas, of consequence for prospective epidemiological or vaccine effectiveness studies using clinical endpoints. Interpretations of the interplay between severity, case definitions and hospitalization are particularly important from a health economics perspective when we consider that a single hospitalization has been reported to cost between USD 289 (Philippines) and USD 863 (Malaysia). [21,46]

The case definition applied in CYD14 (fever ≥ 38°C on ≥ 2 consecutive days) intentionally captured a broad spectrum of disease, enabling calculation of vaccine efficacy against dengue of any severity. However, a considerable proportion of VCD episodes (7.8%) were assessed as DHF by the investigators, and while empirically-derived global burden estimates of severe dengue/DHF are not available, [47] our data suggest that in Southeast Asia, the burden is substantial. Extrapolations using appropriate baselines and harmonized case definitions associated with clinical severity could theoretically be used to generate estimates of severe disease, and may be a topic of further research.

Most clinical diagnoses of dengue were virologically confirmed, resulting in a PPV of 85.2%. However the sensitivity was 28.8%, reflecting the significant proportion of VCD which was not clinically diagnosed as dengue. This is likely because local DF or DHF reporting case definitions had not been satisfied, even when investigators suspected dengue as the underlying aetiology, and is a finding which should contribute to the understanding of the clinical and economic burden of mild dengue disease. Using VCD as a denominator, the complement to clinical diagnoses were termed undifferentiated fever VCD in our study, and represent symptomatic, febrile, virologically confirmed cases which were not diagnosed. Policymakers sometimes consider milder manifestations of disease unimportant, but a recent Cambodian study found mild dengue cases are significantly more infectious than those with symptoms. [48] Mild cases may thus contribute significantly to transmission and constitute an important viral reservoir. Additional studies will be required to understand the impacts on population-level immunity and transmission dynamics.

A clinical diagnostic exclusion of dengue following a febrile episode was correct in >90% of instances (NPV: 92.4%) and the specificity of clinical diagnosis was 99.4% in these epidemiological settings where >10% of acute fevers were caused by dengue virus infection. The accuracy of diagnosis was much improved when considering only hospitalized episodes, indicating that surveillance reports and burden estimates of more severe disease are likely more reliable than those of mild cases. However this leaves a considerable burden of mild disease which is unaccounted for. We are not aware of health economic or healthcare utilization studies examining the impact of these mild episodes but their frequency implies a significant source of burden. Additional analyses could consider aggregating costs (including indirect costs), disability-adjusted life years or other measures to quantify impacts.

The study has limitations. The ID of cVCD was low in some settings, with only six episodes in Malaysia and nine in Vietnam. This is an unavoidable consequence of examining infrequent disease outcomes using prospective methods. Our approach annualized incidence, which may have introduced some bias, but our comparison with local surveillance data and overlapping timeframes will limit geographical/temporal distortions. As this was a vaccine trial, sites were chosen for their historically high reported dengue burdens, so results from lower-endemic areas may differ. [38] For this reason, incidence rates and other findings could not be combined between countries. However, the socio-environmental determinants of dengue incidence are poorly understood and in many Asian countries burdens are unpredictable throughout urban endemic areas. Where age-stratified incidence data were unavailable, adjustments were made which introduced slight inaccuracies to the data. More substantial variability was caused by the differences in national surveillance systems, with more sensitive surveillance giving rise to lower EFs. This is an inherent study bias but also an interesting result; the use of a stable denominator in expansion factor calculations provides an insight into surveillance system specificities.

WHO 1997 classifications were applied, as assessed by investigators, because at study initiation 2009 guidelines were not in routine use at all sites. Cases were also classified according to a more inclusive definition of severe dengue, integrating criteria from the WHO 1997 and 2009 and South East Asia Regional Office 2011 guidelines, and applied by the study Independent Data Monitoring Committee (IDMC). [49] Of the 25 VCD cases clinically diagnosed as DHF, 20 met these IDMC criteria indicating, in this clinical trial environment at least, a level of concordance between the two.

This analysis was performed to inform policy making and strengthen evidence for public health decisions, including financing for dengue control efforts such as vaccination. It adds to available evidence indicating that passive surveillance systems greatly underestimate dengue burden and emphasizes that burden estimates are highly sensitive to case definitions. The control arms of vaccine clinical trials can provide valuable data to estimate disease burdens.

## Supporting Information

S1 Checklist STROBE checklist. (DOC)

S1 File National dengue surveillance system summaries. (DOCX)

S1 Table Characteristics of the control group of the CYD14 study, described by age group, site, and the numbers of dengue and associated clinical episodes occurring during the timeframe of the study. (DOCX)

S2 Table Dengue surveillance data from the areas in which the CYD14 clinical trial was conducted, over the period of study and population data. (DOCX)
