# Supplementary material for: Symptomatic Dengue Disease in Five Southeast Asian Countries: Epidemiological Evidence from a Dengue Vaccine Trial
Source: PLoS Negl Trop Dis. 2016 Aug 17;10(8):e0004918. doi: 10.1371/journal.pntd.0004918 (PMC4988713; doi:10.1371/journal.pntd.0004918)
Supplement: S1 Checklist — (DOC) [file pntd.0004918.s001.doc]

**S1 Checklist.** STROBE Statement—Checklist of items that should be included in reports of ***cohort studies***

|  | Item No | Recommendation |
| --- | --- | --- |
| **Title and abstract** | 1 | (*a*) Indicate the study’s design with a commonly used term in the title or the abstract **[This is an epidemiological analysis of data from a vaccine trial]** |
|  |  | (*b*) Provide in the abstract an informative and balanced summary of what was done and what was found **[Summary and results are described in the abstract]** |
| Introduction | | |
| Background/rationale | 2 | Explain the scientific background and rationale for the investigation being reported **[This is described in the introduction, page 5]** |
| Objectives | 3 | State specific objectives, including any prespecified hypotheses **[This was an exploratory, secondary analysis, as described at the end of the introduction]** |
| Methods | | |
| Study design | 4 | Present key elements of study design early in the paper **[This is described in materials and methods section *CYD14 study design and data*, in addition to a reference to the original vaccine clinical trial]** |
| Setting | 5 | Describe the setting, locations, and relevant dates, including periods of recruitment, exposure, follow-up, and data collection **[Information is within materials and methods section *CYD14 study design and data* in supplementary table 1]** |
| Participants | 6 | (*a*) Give the eligibility criteria, and the sources and methods of selection of participants. Describe methods of follow-up **[The details are provided in the original reference #23]** |
|  |  | (*b*) For matched studies, give matching criteria and number of exposed and unexposed **[N/A]** |
| Variables | 7 | Clearly define all outcomes, exposures, predictors, potential confounders, and effect modifiers. Give diagnostic criteria, if applicable **[The main outcomes are laboratory and clinically diagnosed dengue as described in materials and method *CYD14 study design and data* and Fig 1. WHO diagnostic criteria are described. Confounding was not adjusted for and is discussed in limitations of the discussion, page 21]** |
| Data sources/ measurement | 8* | For each variable of interest, give sources of data and details of methods of assessment (measurement). Describe comparability of assessment methods if there is more than one group **[Study involved comparisons of overlapping data sources, as described in material and methods. The methods of assessment are described]** |
| Bias | 9 | Describe any efforts to address potential sources of bias **[direct standardization was used to control for bias associated with study/census population sizes and the respective dengue epidemiology in each study site. N/A for other aspects of this secondary analysis. ]** |
| Study size | 10 | Explain how the study size was arrived at **[n/a for this secondary analysis]** |
| Quantitative variables | 11 | Explain how quantitative variables were handled in the analyses. If applicable, describe which groupings were chosen and why **[Described in materials and methods *National dengue surveillance, population data and incidence rates*, *Calculating CYD14 incidence densities* and *Expansion factors and case definitions*]** |
| Statistical methods | 12 | (*a*) Describe all statistical methods, including those used to control for confounding |
|  |  | (*b*) Describe any methods used to examine subgroups and interactions |
|  |  | (*c*) Explain how missing data were addressed |
|  |  | (*d*) If applicable, explain how loss to follow-up was addressed |
|  |  | (*e*) Describe any sensitivity analyses  **[Statistical methods and missing data are addressed in section *National dengue surveillance, population data and incidence rates*, *Calculating CYD14 incidence densities.* Direct standardization was applied.]** |
| Results | | |
| Participants | 13* | (a) Report numbers of individuals at each stage of study—eg numbers potentially eligible, examined for eligibility, confirmed eligible, included in the study, completing follow-up, and analysed |
|  |  | (b) Give reasons for non-participation at each stage |
|  |  | (c) Consider use of a flow diagram  **[Presented in results section *VCD and CDD in the CYD14 cohorts* and the number of episodes in Fig 1.]** |
| Descriptive data | 14* | (a) Give characteristics of study participants (eg demographic, clinical, social) and information on exposures and potential confounders |
|  |  | (b) Indicate number of participants with missing data for each variable of interest |
|  |  | (c) Summarise follow-up time (eg, average and total amount)  **[A full description of subject demographics is provided in refs [23] and [24]. Total follow-up time is presented in results]** |
| Outcome data | 15* | Report numbers of outcome events or summary measures over time  **[Presented in results, tables 1 – 4]** |
| Main results | 16 | (*a*) Give unadjusted estimates and, if applicable, confounder-adjusted estimates and their precision (eg, 95% confidence interval). Make clear which confounders were adjusted for and why they were included **[Adjusting was only performed by directl standardization as described in materials and methods]** |
|  |  | (*b*) Report category boundaries when continuous variables were categorized **[N/A]** |
|  |  | (*c*) If relevant, consider translating estimates of relative risk into absolute risk for a meaningful time period **[N/A]** |
| Other analyses | 17 | Report other analyses done—eg analyses of subgroups and interactions, and sensitivity analyses **[N/A, all analyses are reported]** |
| Discussion | | |
| Key results | 18 | Summarise key results with reference to study objectives **[addressed throughout discussion]** |
| Limitations | 19 | Discuss limitations of the study, taking into account sources of potential bias or imprecision. Discuss both direction and magnitude of any potential bias **[Discussed on page 21]** |
| Interpretation | 20 | Give a cautious overall interpretation of results considering objectives, limitations, multiplicity of analyses, results from similar studies, and other relevant evidence **[Interpretation is aligned with results and existing literature]** |
| Generalisability | 21 | Discuss the generalisability (external validity) of the study results **[Generalizibility of results is limited as discussed on page 21, but concepts are generalizable, and implication are discussed throughout the discussion section.]** |
| Other information | | |
| Funding | 22 | Give the source of funding and the role of the funders for the present study and, if applicable, for the original study on which the present article is based **[N/A; this was a secondary analysis which received no funding. Affiliations of all authors are shared. Funded of the original vaccine trial is described in reference [23]]** |

*Give information separately for exposed and unexposed groups.

**Note:** An Explanation and Elaboration article discusses each checklist item and gives methodological background and published examples of transparent reporting. The STROBE checklist is best used in conjunction with this article (freely available on the Web sites of PLoS Medicine at http://www.plosmedicine.org/, Annals of Internal Medicine at http://www.annals.org/, and Epidemiology at http://www.epidem.com/). Information on the STROBE Initiative is available at http://www.strobe-statement.org.
