# Supplementary material for: Symptomatic Dengue Disease in Five Southeast Asian Countries: Epidemiological Evidence from a Dengue Vaccine Trial
Source: PLoS Negl Trop Dis. 2016 Aug 17;10(8):e0004918. doi: 10.1371/journal.pntd.0004918 (PMC4988713; doi:10.1371/journal.pntd.0004918)
Supplement: S1 File — (DOCX) [file pntd.0004918.s002.docx]

**S1 File. National dengue surveillance system summaries**

**Indonesia**

Reporting of DHF in Indonesia is mandatory within 72 hours of diagnosis but is acknowledged by Indonesian experts to be incomplete and to vary widely between provinces and islands. Routine passive surveillance reports from sub-district health centers (*Puskesmas*) or other healthcare facilities are aggregated at district/municipal health offices and compiled weekly at provincial and national levels, with corresponding feedback to inform outbreak responses [1]. During outbreaks, the frequency of reporting may be increased[2]. Laboratory confirmation of dengue is rare, especially in health services with limited facilities. Diagnoses therefore tend to be based on clinical criteria accompanied by basic hematology laboratory results. Health centers and public/private hospitals continue to use 1997 WHO case definitions: only DHF/DSS cases should be reported. Genotypic and serological surveillance has been undertaken on a project basis by some Indonesian institutions [3–5]. Surveillance data at the national level are not reported publically; Jakarta municipality maintains an epidemiological surveillance website [6].

**Malaysia**

Malaysia’s first recorded dengue cases were in 1901, in Penang, and the disease has since become endemic nationwide [7]. Dengue suspected and confirmed case reporting is mandatory within 24 hours, both from inpatient and outpatient settings, to the district health office [8,9]. In 2010, the surveillance system was updated to the real-time, electronic “eDengue” system, enabling prompt and automated outbreak response [10]. Serotype confirmation is conducted on a subset of clinical samples from sentinel sites, allowing monitoring of both serotypes and genotypes and the country contributes to UNITEDengue, a regional surveillance and laboratory confirmation network [11]. Reporting is according to clinical and laboratory diagnosis and since 2014, rapid diagnostic tests have been available at public health facilities, increasing the number and proportion of confirmed cases in the country [12]. A combination of WHO 1997 and 2009 case definitions are applied clinically and in the surveillance system, where data are publically available [13,14].

**Philippines**

Dengue has been a notifiable disease in the Philippines since 1958, in 2008 transitioning from a sentinel (National Epidemic Sentinel Surveillance System) to an all-case reporting system (Philippines Integrated Disease Surveillance and Response System [PIDSR]), with >1,500 disease reporting units [15]. Suspected, probable, or confirmed cases are electronically reported at health centers, district, provincial and regional hospitals and aggregated at sub-national and national levels, and publically disseminated[16]. Dengue incidence is particularly high in regions NCR, I, III, Iva, VI, VII, and XI (average incidence rate 2010 - 2014: 188.4/100,000), and most cases are <20 years old [17]. Both the 1997 and 2009 WHO classification systems are used for dengue clinical management and case reporting [15,18]. A sub-sample of suspected cases is submitted to a national reference laboratory for laboratory confirmation by RT-PCR and by IgG and IgM ELISA. In 2014, the Philippines initiated systematic serotype surveillance at 20 sentinel hospitals [10,18].

**Thailand**

Dengue was first reported in Thailand in 1949 and was followed in 1958 by the first DHF epidemic, after which disease reporting became mandatory [19]. The passive dengue surveillance system was functioning by 1974, and is augmented by active components (case investigation; vector/virus surveillance) during outbreaks [10,20]. Suspected, probable and confirmed cases according to adapted WHO 1997 case definitions must be reported within 24 hours to the district health offices after which they are aggregated at provincial health offices and the Bureau of Epidemiology [10,21,22]. The system is supervised by five Regional Epidemiological Centers to improve accuracy [20]. Electronic or paper report forms contain the age, sex, day of onset, and address where the case occurred [20]. Summaries are published on the Bureau of Epidemiology website [23]. Nearly 90% of cases are reported from public health facilities, mostly from community hospitals, general hospitals, and regional hospitals [24].

**Vietnam**

Dengue surveillance is conducted by The National Program for Dengue Control, which was established following an outbreak in 1998 [25]. The program is supported by four regional institutes that provide technical and laboratory support: the National Institute for Hygiene and Epidemiology (NIHE) in Hanoi (Northern), the Tay Nguyen Institute for Hygiene and Epidemiology (Central Highlands), the Nha Trang Pasteur Institute (Central), and the Ho Chi Minh Pasteur Institute (Southern). The surveillance system is designed to detect dengue cases at the commune health service level in a timely (≤ 24 hours) and standardized manner. Since 2011, the WHO 2009 dengue classification has been used for case management and reporting [26,27]. Data are predominantly from inpatient clinics of public hospitals, and cases from private facilities are under-reported [10]. Data are aggregated according to severity and to two age groups (≤15 years old and >15 years old) and regularly reported to lower-level healthcare systems [10,28]. Viral surveillance is conducted in sentinel hospitals and from routine surveillance: 10% of confirmed cases are serotyped and 3% are confirmed by virus isolation [10,25].

**References**

1. Arbovirosis Control Subdirectorate VBDC Directorate Indonesia MoH. Dengue Surveillance Presentation, Workshop in Yogyakarta. 2013.

2. Buletin Jendela Epidemiologi , Topik utama: demam berdarah dengue. 2010;2(August). Available from: http://www.depkes.go.id/downloads/publikasi/buletin/BULETIN DBD.pdf

3. Fahri S, Yohan B, Trimarsanto H, Sayono S, Hadisaputro S, Dharmana E, et al. Molecular surveillance of dengue in semarang, indonesia revealed the circulation of an old genotype of dengue virus serotype-1. PLoS Negl Trop Dis [Internet]. 2013 Aug [cited 2013 Aug 30];7(8):e2354. Available from: http://www.pubmedcentral.nih.gov/articlerender.fcgi?artid=3738473&tool=pmcentrez&rendertype=abstract

4. Porter KR, Beckett CG, Kosasih H, Tan RI, Alisjahbana B, Rudiman PIF, et al. Epidemiology of dengue and dengue hemorrhagic fever in a cohort of adults living in Bandung, West Java, Indonesia. Am J Trop Med Hyg [Internet]. 2005 Jan;72(1):60–6. Available from: http://www.ncbi.nlm.nih.gov/pubmed/15728868

5. Setiati TE, Wagenaar JFP, Kruif MD De, Mairuhu ATA. Changing Epidemiology of Dengue Haemorrhagic Fever in Indonesia. 2006;30:1–14.

6. Jakarta Health Department. Disease Surveillance Portal [Internet]. [cited 2015 May 1]. Available from: http://www.surveilans-dinkesdki.net/

7. Poovaneswari S. Dengue situation in Malaysia. Malays J Pathol [Internet]. 1993 Jun [cited 2015 Aug 5];15(1):3–7. Available from: http://www.ncbi.nlm.nih.gov/pubmed/8277787

8. Mohd-Zaki AH, Brett J, Ismail E, L’Azou M. Epidemiology of Dengue Disease in Malaysia (2000–2012): A Systematic Literature Review. Horstick O, editor. PLoS Negl Trop Dis [Internet]. 2014 Nov 6 [cited 2014 Nov 7];8(11):e3159. Available from: http://dx.plos.org/10.1371/journal.pntd.0003159

9. Beatty ME, Stone A, Fitzsimons DW, Hanna JN, Lam SK, Vong S, et al. Best practices in dengue surveillance: a report from the Asia-Pacific and Americas Dengue Prevention Boards. PLoS Negl Trop Dis [Internet]. 2010 Jan [cited 2014 Aug 9];4(11):e890. Available from: http://www.pubmedcentral.nih.gov/articlerender.fcgi?artid=2982842&tool=pmcentrez&rendertype=abstract

10. ASEAN Dengue Vaccine Advocacy. Report of 1st ADVA Regional Workshop. Bangkok; 2012.

11. Ng L-C, Chem Y-K, Koo C, Mudin RNB, Amin FM, Lee K-S, et al. 2013 Dengue Outbreaks in Singapore and Malaysia Caused by Different Viral Strains. Am J Trop Med Hyg [Internet]. 2015 Apr 6 [cited 2015 May 5]; Available from: http://www.ncbi.nlm.nih.gov/pubmed/25846296

12. Malaysia Ministry of Health, personal communication.

13. Ministry of Health Malaysia. Weekly Dengue Epidemiological Update [Internet]. Available from: http://www.moh.gov.my/index.php/database_stores/store_view/17

14. Sam S-S, Omar SFS, Teoh B-T, Abd-Jamil J, AbuBakar S. Review of Dengue hemorrhagic fever fatal cases seen among adults: a retrospective study. PLoS Negl Trop Dis [Internet]. 2013 Jan [cited 2014 Jul 25];7(5):e2194. Available from: http://www.pubmedcentral.nih.gov/articlerender.fcgi?artid=3642057&tool=pmcentrez&rendertype=abstract

15. Bravo L, Roque VG, Brett J, Dizon R, L’Azou M. Epidemiology of Dengue Disease in the Philippines (2000–2011): A Systematic Literature Review. Horstick O, editor. PLoS Negl Trop Dis [Internet]. 2014 Nov 6 [cited 2014 Nov 7];8(11):e3027. Available from: http://dx.plos.org/10.1371/journal.pntd.0003027

16. Philippines DoH National Epidemiology Centre. National Epidemiology Centre Dengue Surveillance Reports. Available from: http://www.nec.doh.gov.ph/index.php?option=com_content&view=article&id=68&Itemid=92

17. Philippines DoH National Epidemiology Centre. National dengue surveillance data. 2014.

18. Philippines Department of Health, personal communication.

19. Limkittikul K, Brett J, L’Azou M. Epidemiological Trends of Dengue Disease in Thailand (2000–2011): A Systematic Literature Review. Halstead SB, editor. PLoS Negl Trop Dis [Internet]. 2014 Nov 6 [cited 2014 Nov 7];8(11):e3241. Available from: http://dx.plos.org/10.1371/journal.pntd.0003241

20. Chareonsook O, Foy HM, Teeraratkul a, Silarug N. Changing epidemiology of dengue hemorrhagic fever in Thailand. Epidemiol Infect [Internet]. 1999 Feb;122(1):161–6. Available from: http://www.pubmedcentral.nih.gov/articlerender.fcgi?artid=2809602&tool=pmcentrez&rendertype=abstract

21. Thaewnongiew K, Promthet S, Nilvarangkul K, Rangsin R, Phitak P, Sarakarn P. The surveillance system in health centers in northeastern Thailand. Jpn J Infect Dis [Internet]. 2009 Nov [cited 2015 Aug 5];62(6):444–9. Available from: http://www.ncbi.nlm.nih.gov/pubmed/19934536

22. Wattanasri S. Presentation: Communicable Disease Surveillance [Internet]. Available from: file:///C:/Users/I0193603/Downloads/COMUNIC- DISEASE SURVEILLANCE.pdf

23. Thailand Ministry of Public Health. Thai Bureau of Epidemiology [Internet]. Available from: http://203.157.15.110/boe/home.php#

24. Muangchana C. Epidemiology of Dengue in Thailand & Implications of Vaccine Introduction on Vector Control. Brazilia; 2013.

25. National Institute of Hygiene and Epidemiology. Dengue Haemorrhagic Fever Establishment History [Internet]. Available from: http://www.nihe.org.vn/new-en/chuong-trinh-giam-sat-theo-doi-nhiem-hivaids/1091/Establishment-History.vhtm

26. Arima Y, Matsui T. Epidemiologic update of dengue in the Western Paciﬁc Region, 2010. West Pacific Surveill Response J. 2011;2(2):1–7.

27. Badurdeen S, Valladares DB, Farrar J, Gozzer E, Kroeger A, Kuswara N, et al. Sharing experiences: towards an evidence based model of dengue surveillance and outbreak response in Latin America and Asia. BMC Public Health [Internet]. BMC Public Health; 2013 Jan [cited 2013 Jul 31];13(1):607. Available from: http://www.pubmedcentral.nih.gov/articlerender.fcgi?artid=3697990&tool=pmcentrez&rendertype=abstract

28. Kindly provided by Ministry of Health Department of Preventive Medicine Ho Chi Minh. Dengue situation in 2014 & key actions for 2015. 2015.
