# Supplementary material for: Symptomatic Dengue Disease in Five Southeast Asian Countries: Epidemiological Evidence from a Dengue Vaccine Trial
Source: PLoS Negl Trop Dis. 2016 Aug 17;10(8):e0004918. doi: 10.1371/journal.pntd.0004918 (PMC4988713; doi:10.1371/journal.pntd.0004918)
Supplement: S1 Table — (DOCX) [file pntd.0004918.s003.docx]

**S1 Table.** Characteristics of the control group of the CYD14 study, described by age group, site, and the numbers of dengue and associated clinical episodes occurring during the timeframe of the study.

| **Country and site** | **Dengue data from CYD14 clinical trial control group** | | | | | | | | | | | | | | | | | | |
| --- | --- | --- | --- | --- | --- | --- | --- | --- | --- | --- | --- | --- | --- | --- | --- | --- | --- | --- | --- |
|  | **age group** | **n, injected** | **n, person-years followed** | **n, febrile episodes** | **n, VCD** | **n, hospitalizations** | | **n, clinically diagnosed as dengue, hospitalized** | | **n, clinically diagnosed as dengue, non hospitalized** | | **n, clinically diagnosed DHF, hospitalized** | | **n, clinically diagnosed DHF, non hospitalized** | | **ID, per 100 person-years** | | | |
|  |  |  |  |  |  | **VCD** | **not VCD** | **VCD** | **not VCD** | **VCD** | **not VCD** | **VCD** | **not VCD** | **VCD** | **not VCD** | **VCD** | **cVCD** | **CDD** | **UF-VCD** |
| **Indonesia** |  | **623** | **1,232** | **357** | **44** | **20** | **10** | **19** | **2** | **7** | **5** | **10** | **1** | **0** | **0** | **3.571** | **2.110** | **2.679** | **1.461** |
| Jakarta | 2 - 14 | 250 | 495 | 117 | 12 | 6 | 2 | 6 | 0 | 2 | 3 | 4 | 0 | 0 | 0 | 2.424 | 1.616 | 2.222 | 0.808 |
|  | < 5 | 34 | 41 | 22 | 0 | 0 | 0 | 0 | 0 | 0 | 0 | 0 |  |  |  | 0.000 | 0.000 | 0.000 | 0.000 |
|  | 5 - <10 | 113 | 197 | 58 | 8 | 3 | 2 | 3 | 0 | 2 | 1 | 2 |  |  |  | 4.061 | 2.538 | 3.046 | 1.523 |
|  | >10 | 103 | 258 | 37 | 4 | 3 | 0 | 3 | 0 | 0 | 2 | 2 |  |  |  | 1.550 | 1.163 | 1.938 | 0.388 |
| Bandung | 2 - 14 | 247 | 491 | 151 | 22 | 9 | 5 | 8 | 1 | 3 | 1 | 4 | 0 | 0 | 0 | 4.481 | 2.240 | 2.648 | 2.240 |
|  | < 5 | 49 | 55 | 24 | 4 | 2 | 0 | 2 | 0 | 1 | 1 | 0 |  |  |  | 7.273 | 5.455 | 7.273 | 1.818 |
|  | 5 - <10 | 162 | 299 | 97 | 17 | 7 | 4 | 6 | 1 | 2 | 0 | 4 |  |  |  | 5.686 | 2.676 | 3.010 | 3.010 |
|  | >10 | 36 | 136 | 30 | 1 | 0 | 1 | 0 | 0 | 0 | 0 | 0 |  |  |  | 0.735 | 0.000 | 0.000 | 0.735 |
| Denpasar | 2 - 14 | 126 | 246 | 89 | 10 | 5 | 3 | 5 | 1 | 2 | 1 | 2 | 1 | 0 | 0 | 4.065 | 2.846 | 3.659 | 1.220 |
|  | < 5 | 13 | 14 | 12 | 1 | 0 | 1 | 0 | 1 | 0 | 0 | 0 | 1 |  |  | 7.143 | 0.000 | 7.143 | 7.143 |
|  | 5 - <10 | 60 | 104 | 46 | 3 | 0 | 0 | 0 | 0 | 2 | 0 | 0 | 0 |  |  | 2.885 | 1.923 | 1.923 | 0.962 |
|  | >10 | 53 | 128 | 31 | 6 | 5 | 2 | 5 | 0 | 0 | 1 | 2 | 0 |  |  | 4.688 | 3.906 | 4.688 | 0.781 |
| **Malaysia** |  | **465** | **937** | **332** | **21** | **8** | **12** | **7** | **0** | **2** | **2** | **1** | **0** | **0** | **0** | **2.241** | **0.961** | **1.174** | **1.281** |
| Kuala Lumpur and Putrajaya | 2 - 14 | 309 | 624 | 288 | 17 | 5 | 6 | 5 | 0 | 2 | 2 | 0 | 0 | 0 | 0 | 2.724 | 1.122 | 1.442 | 1.603 |
|  | < 5 | 54 | 60 | 54 | 2 | 0 | 0 | 0 | 0 | 0 | 0 |  |  |  |  | 3.333 | 0.000 | 0.000 | 3.333 |
|  | 5 - <10 | 130 | 257 | 156 | 7 | 3 | 6 | 3 | 0 | 1 | 0 |  |  |  |  | 2.724 | 1.556 | 1.556 | 1.167 |
|  | >10 | 125 | 307 | 78 | 8 | 2 | 0 | 2 | 0 | 1 | 2 |  |  |  |  | 2.606 | 0.977 | 1.629 | 1.629 |
| Penang | 2 - 14 | 156 | 313 | 44 | 4 | 3 | 6 | 2 | 0 | 0 | 0 | 1 | 0 | 0 | 0 | 1.278 | 0.639 | 0.639 | 0.639 |
|  | < 5 | 20 | 21 | 3 | 0 | 0 | 1 | 0 | 0 |  |  | 0 |  |  |  | 0.000 | 0.000 | 0.000 | 0.000 |
|  | 5 - <10 | 59 | 109 | 18 | 3 | 2 | 3 | 1 | 0 |  |  | 1 |  |  |  | 2.752 | 0.917 | 0.917 | 1.835 |
|  | >10 | 77 | 183 | 23 | 1 | 1 | 2 | 1 | 0 |  |  | 0 |  |  |  | 0.546 | 0.546 | 0.546 | 0.000 |
| **Philippines** |  | **1,166** | **2,370** | **1,420** | **156** | **17** | **18** | **15** | **2** | **1** | **1** | **9** | **1** | **0** | **0** | **6.582** | **0.675** | **0.802** | **5.907** |
| San Pablo City | 2 - 14 | 855 | 1754 | 773 | 82 | 12 | 15 | 11 | 2 | 0 | 1 | 6 | 1 | 0 | 0 | 4.675 | 0.627 | 0.798 | 4.048 |
|  | < 5 | 198 | 253 | 178 | 7 | 0 | 4 | 0 | 0 |  | 0 | 0 | 0 |  |  | 2.767 | 0.000 | 0.000 | 2.767 |
|  | 5 - <10 | 304 | 651 | 338 | 35 | 6 | 6 | 6 | 1 |  | 1 | 3 | 0 |  |  | 5.376 | 0.922 | 1.229 | 4.455 |
|  | >10 | 353 | 850 | 257 | 40 | 6 | 5 | 5 | 1 |  | 0 | 3 | 1 |  |  | 4.706 | 0.588 | 0.706 | 4.118 |
| Cebu | 2 - 14 | 311 | 616 | 647 | 74 | 5 | 3 | 4 | 0 | 1 | 0 | 3 | 0 | 0 | 0 | 12.013 | 0.812 | 0.812 | 11.201 |
|  | < 5 | 75 | 96 | 166 | 15 | 0 | 2 | 0 | 0 | 0 |  | 0 |  |  |  | 15.625 | 0.000 | 0.000 | 15.625 |
|  | 5 - <10 | 123 | 231 | 281 | 32 | 4 | 1 | 3 | 0 | 0 |  | 2 |  |  |  | 13.853 | 1.299 | 1.299 | 12.554 |
|  | >10 | 113 | 289 | 200 | 27 | 1 | 0 | 1 | 0 | 1 |  | 1 |  |  |  | 9.343 | 0.692 | 0.692 | 8.651 |
| **Thailand** |  | **392** | **792** | **388** | **47** | **13** | **21** | **12** | **1** | **23** | **0** | **2** | **0** | **0** | **0** | **5.934** | **4.419** | **4.545** | **1.515** |
| Kamphaeng Phet | 2 - 14 | 196 | 397 | 217 | 31 | 8 | 6 | 8 | 0 | 19 | 0 | 2 | 0 | 0 | 0 | 7.809 | 6.801 | 6.801 | 1.008 |
|  | < 5 | 41 | 52 | 66 | 2 | 0 | 2 | 0 | 0 | 2 |  | 0 |  |  |  | 3.846 | 3.846 | 3.846 | 0.000 |
|  | 5 - <10 | 64 | 141 | 94 | 15 | 4 | 4 | 4 | 0 | 9 |  | 1 |  |  |  | 10.638 | 9.220 | 9.220 | 1.418 |
|  | >10 | 91 | 204 | 57 | 14 | 4 | 0 | 4 | 0 | 8 |  | 1 |  |  |  | 6.863 | 5.882 | 5.882 | 0.980 |
| Ratchaburi | 2 - 14 | 196 | 395 | 171 | 16 | 5 | 15 | 4 | 1 | 4 | 0 | 0 | 0 | 0 | 0 | 4.051 | 2.025 | 2.278 | 2.025 |
|  | < 5 | 37 | 43 | 44 | 3 | 1 | 2 | 0 | 0 | 1 |  |  |  |  |  | 6.977 | 2.326 | 2.326 | 4.651 |
|  | 5 - <10 | 88 | 180 | 87 | 8 | 2 | 8 | 2 | 0 | 2 |  |  |  |  |  | 4.444 | 2.222 | 2.222 | 2.222 |
|  | >10 | 71 | 172 | 40 | 5 | 2 | 5 | 2 | 1 | 1 |  |  |  |  |  | 2.907 | 1.744 | 2.326 | 1.163 |
| **Vietnam** |  | **778** | **1,602** | **602** | **51** | **3** | **4** | **3** | **1** | **3** | **2** | **2** | **1** | **1** | **1.00** | **3.184** | **0.375** | **0.562** | **2.809** |
| Long Xuyen | 2 - 14 | 467 | 957 | 356 | 24 | 2 | 0 | 2 | 0 | 1 | 1 | 2 | 0 | 1 | 0 | 2.508 | 0.313 | 0.418 | 2.194 |
|  | < 5 | 33 | 41 | 40 | 0 | 0 | 0 | 0 | 0 | 0 | 1 | 0 |  | 0 |  | 0.000 | 0.000 | 2.439 | 0.000 |
|  | 5 - <10 | 207 | 348 | 155 | 9 | 1 | 0 | 1 | 0 | 0 | 0 | 1 |  | 0 |  | 2.586 | 0.287 | 0.287 | 2.299 |
|  | >10 | 227 | 568 | 161 | 15 | 1 | 0 | 1 | 0 | 1 | 0 | 1 |  | 1 |  | 2.641 | 0.352 | 0.352 | 2.289 |
| My Tho | 2 - 14 | 311 | 645 | 246 | 27 | 1 | 4 | 1 | 1 | 2 | 1 | 0 | 1 | 0 | 1 | 4.186 | 0.465 | 0.775 | 3.721 |
|  | < 5 | 34 | 41 | 28 | 2 | 0 | 2 | 0 | 0 | 0 | 0 |  | 0 |  | 0 | 4.878 | 0.000 | 0.000 | 4.878 |
|  | 5 - <10 | 161 | 296 | 129 | 12 | 1 | 2 | 1 | 1 | 1 | 0 |  | 1 |  | 0 | 4.054 | 0.676 | 1.014 | 3.378 |
|  | >10 | 116 | 308 | 89 | 13 | 0 | 0 | 0 | 0 | 1 | 1 |  | 0 |  | 1 | 4.221 | 0.325 | 0.649 | 3.896 |

ID, incidence density; CDD, clinically diagnosed dengue; cVCD, clinically diagnosed and virologically confirmed dengue; DHF, dengue hemorrhagic fever; ID, incidence density; n, number of subjects or events; UF-VCD, virologically confirmed dengue clinically diagnosed as undifferentiated fever; VCD, virologically confirmed dengue.
