# Supplementary material for: Symptomatic Dengue Disease in Five Southeast Asian Countries: Epidemiological Evidence from a Dengue Vaccine Trial
Source: PLoS Negl Trop Dis. 2016 Aug 17;10(8):e0004918. doi: 10.1371/journal.pntd.0004918 (PMC4988713; doi:10.1371/journal.pntd.0004918)
Supplement: S2 Table — (DOCX) [file pntd.0004918.s004.docx]

**S2 Table.** Dengue surveillance data from the areas in which the CYD14 clinical trial was conducted, over the period of study and population data (for specifics, see material and methods). Direct standardization was used to calculate age and site adjusted incidence rates from the CYD14 cohort (see S2 table for unadjusted rates).

| **Country and site** | **Dengue data reported from national routine surveillance** | | | | | | **Age-and site-adjusted incidence, cases per 100,000 person-years** | | | |
| --- | --- | --- | --- | --- | --- | --- | --- | --- | --- | --- |
|  | **Age group** | **n, cases over the study period** | **Population (mid study point)** | **Average annual IR, cases per 100/year** | **Weight by direct standardization** | | **VCD** | **cVCD** | **CDD** | **UF-VCD** |
|  |  |  |  |  | **Site** | **Age** |  |  |  |  |
| **Indonesia** |  | **26,375** | **4,013,640** | **0.263** |  |  | **3,017** | **1,825** | **2,479** | **1,192** |
| Jakarta | <15 | 13,773 | 2,408,900 | 0.229 | 0.60 |  | 1,827 | 1,201 | 1,604 | 627 |
|  | <5 | 4,582 | 876,039 | 0.209 |  | 0.36 | 0 | 0 | 0 | 0 |
|  | 5 - 9 | 4,845 | 806,753 | 0.240 |  | 0.33 | 1,360 | 850 | 1,020 | 510 |
|  | 10 - 14 | 4,346 | 726,108 | 0.239 |  | 0.30 | 467 | 350 | 584 | 117 |
| Bandung | <15 | 11,817 | 1,397,387 | 0.338 | 0.35 |  | 4,771 | 2,880 | 3,653 | 1,891 |
|  | <5 | 3,931 | 508,184 | 0.309 |  | 0.36 | 2,645 | 1,984 | 2,645 | 661 |
|  | 5 - 9 | 4,157 | 467,992 | 0.355 |  | 0.33 | 1,904 | 896 | 1,008 | 1,008 |
|  | 10 - 14 | 3,729 | 421,211 | 0.354 |  | 0.30 | 222 | 0 | 0 | 222 |
| Denpasar | <15 | 785 | 207,354 | 0.151 | 0.05 |  | 5,010 | 1,972 | 4,729 | 3,039 |
|  | <5 | 159 | 71,857 | 0.088 |  | 0.35 | 2,475 | 0 | 2,475 | 2,475 |
|  | 5 - 14 | 626 | 135,497 | 0.185 |  | 0.65 | 2,535 | 1,972 | 2,253 | 563 |
|  |  |  |  |  |  |  | 0 | 0 | 0 | 0 |
| **Malaysia** |  | **1,172** | **750,288** | **0.062** |  |  | **2,048** | **671** | **777** | **1,377** |
| Kuala Lumpur and Putrajaya | 0-14 | 885 | 393,326 | 0.090 | 0.52 |  | 2,902 | 829 | 1031 | 2,073 |
|  | 0-4 | 163 | 138,376 | 0.047 |  | 0.35 | 1173 | 0 | 0 | 1,173 |
|  | 5-9 | 220 | 132,793 | 0.066 |  | 0.34 | 920 | 525 | 525 | 394 |
|  | 10-14 | 502 | 122,157 | 0.164 |  | 0.31 | 809 | 303 | 506 | 506 |
| Penang | 0-14 | 287 | 356,962 | 0.032 | 0.48 |  | 1108 | 497 | 497 | 610 |
|  | 0-4 | 55 | 112,677 | 0.020 |  | 0.32 | 0 | 0 | 0 | 0 |
|  | 5-9 | 79 | 118,723 | 0.027 |  | 0.33 | 915 | 305 | 305 | 610 |
|  | 10-14 | 153 | 125,562 | 0.049 |  | 0.35 | 192 | 192 | 192 | 0 |
| **Philippines** |  | **11,491** | **481,588** | **0.954** |  |  | **10,964** | **677** | **701** | **10,287** |
| San Pablo City | <15 | 2,052 | 86,362 | 0.950 | 0.18 |  | 4,254 | 489 | 622 | 3,765 |
|  | <5 | 418 | 28,765 | 0.581 |  | 0.33 | 922 | 0 | 0 | 922 |
|  | 5 - 14 | 1,634 | 57,597 | 1.135 |  | 0.67 | 3,332 | 489 | 622 | 2,844 |
|  |  |  |  | 0.000 |  |  | 0 | 0 | 0 | 0 |
| Cebu | <20 | 9,439 | 395,226 | 0.955 | 0.82 |  | 12,430 | 718 | 718 | 11,712 |
|  | <5 | 8,170 | 100,106 | 3.265 |  | 0.25 | 3,958 | 0 | 0 | 3,958 |
|  | 5 - 19 | 1,269 | 295,120 | 0.172 |  | 0.75 | 8,472 | 718 | 718 | 7,754 |
|  |  | 0 |  | #DIV/0! |  |  | 0 | 0 | 0 | 0 |
| **Thailand** |  | **2,775** | **248,298** | **0.497** |  |  | **5,938** | **4,262** | **4,383** | **1,676** |
| Kamphaeng Phet | 2-14 | 1,092 | 117,384 | 0.414 | 0.47 |  | 7,651 | 6,721 | 6,721 | 930 |
|  | 2-4 | 99 | 26,404 | 0.167 |  | 0.22 | 865 | 865 | 865 | 0 |
|  | 5-9 | 384 | 45,603 | 0.374 |  | 0.39 | 4133 | 3582 | 3582 | 551 |
|  | 10-14 | 609 | 45,378 | 0.597 |  | 0.39 | 2653 | 2274 | 2274 | 379 |
| Ratchaburi | 2-14 | 1683 | 130,914 | 0.571 | 0.53 |  | 4403 | 2057 | 2286 | 2345 |
|  | 2-4 | 188 | 29064 | 0.287 |  | 0.22 | 1549 | 516 | 516 | 1033 |
|  | 5-9 | 633 | 50439 | 0.558 |  | 0.39 | 1712 | 856 | 856 | 856 |
|  | 10-14 | 862 | 51410 | 0.745 |  | 0.39 | 1142 | 685 | 913 | 457 |
| **Vietnam** |  | **1185** | **103,435** | **0.509** |  |  | **2784** | **261** | **840** | **2523** |
| Long Xuyen | <15 | 592 | 62,655 | 0.420 | 0.61 |  | 1743 | 213 | 1025 | 1530 |
|  |  |  | 20,864 | 0.000 |  | 0.33 | 0 | 0 | 812 | 0 |
|  |  |  | 21,115 | 0.000 |  | 0.34 | 872 | 97 | 97 | 775 |
|  |  |  | 20,676 | 0.000 |  | 0.33 | 871 | 116 | 116 | 755 |
| My Tho | <15 | 593 | 40,780 | 0.646 | 0.39 |  | 4383 | 335 | 556 | 4049 |
|  |  |  | 13,580 | 0.000 |  | 0.33 | 1624 | 0 | 0 | 1624 |
|  |  |  | 13,743 | 0.000 |  | 0.34 | 1366 | 228 | 342 | 1139 |
|  |  |  | 13,457 | 0.000 |  | 0.33 | 1393 | 107 | 214 | 1286 |

IR, incidence rate; CDD, clinically diagnosed dengue; cVCD, clinically diagnosed and virologically confirmed dengue; ID, incidence density; n, number of subjects or events; UF-VCD, virologically confirmed dengue clinically diagnosed as undifferentiated fever; VCD, virologically confirmed dengue.
